# A Novel Analog Interpolation Method for Heterodyne Laser Interferometer

**DOI:** 10.3390/mi14030696

**Published:** 2023-03-21

**Authors:** Chung-Ping Chang, Syuan-Cheng Chang, Yung-Cheng Wang, Pin-Yi He

**Affiliations:** 1 Department of Mechanical and Energy Engineering, National Chiayi University, Chiayi 600, Taiwan; 2 Department of Mechanical Engineering, National Yunlin University of Science and Technology, Yunlin 640, Taiwan

**Keywords:** laser interferometer, interpolation, lock-in amplifier, heterodyne, resolution

## Abstract

Laser interferometer technology is used in the precision positioning stage as an encoder. For better resolution, laser interferometers usually work with interpolation devices. According to the interpolation factor, these devices can convert an orthogonal sinusoidal signal into several square-wave signals via digital processing. The bandwidth of the processing will be the limitation of the moving speed of the positioning stage. Therefore, the user needs to make a trade-off between the interpolation factor and the moving speed. In this investigation, a novel analog interpolation method for a heterodyne laser interferometer has been proposed. This method is based on the principle of the lock-in amplifier (LIA). By using the proposed interpolation method, the bandwidth of the laser encoder system can be independent of the interpolation factor. This will be a significant benefit for the ultra-high resolution encoder system and the laser interferometers. The concept, design, and experiment are revealed in this manuscript. The experimental results show that the proposed interpolation method can reach nanometer resolution with a heterodyne laser interferometer, and the bandwidth of the signal is independent of the resolution.

## 1. Introduction

Precision positioning technology is one of the most important techniques in the modern manufacturing industry, and the encoding system is the key to it. Advanced encoder systems are usually based on laser interferometers, due to their high initial resolution and the connection to the definition of the meter [1]. The common initial resolutions of the laser interferometers are about 0.32 μm or 0.16 μm, depending on the optical structure [2] (Table S1, Supplementary Material). The initial resolution of the laser interferometer is several times higher than that of a grating-based encoder system [3], and laser interferometers also have the feature of error reduction [4,5,6]. Therefore, the interferometry−based encoders have high potential in precision positioning.

**Figure 1:**
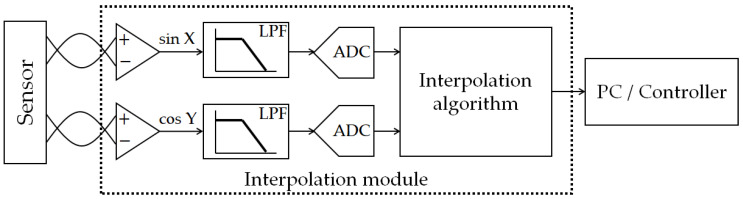
Block diagram of the interpolation module.

**Figure 2:**
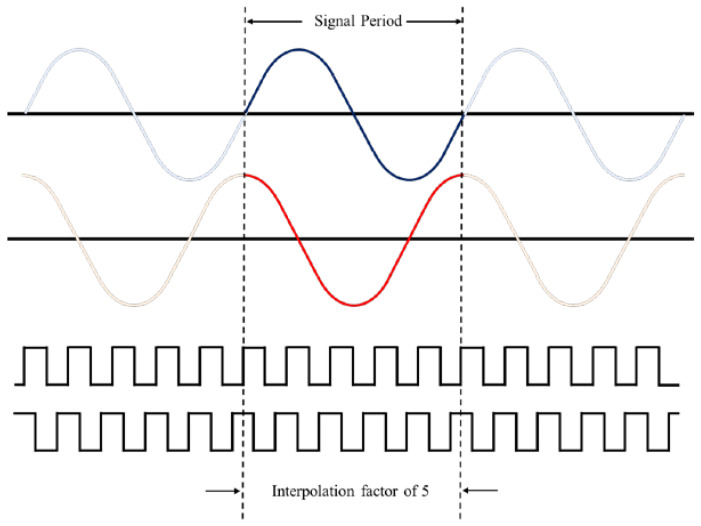
Schematic diagram of interpolation signal converting.

However, the initial resolution of the interferometer systems is on a sub-micrometer scale, which is far from enough for the precision positioning. Therefore, interpolation technology is applied for better resolutions [7]. From the previous research, the interpolation technologies are based on digital signal processing, especially the analog-to-digital converter (ADC), processing algorithms, and microcontroller unit (MCU) [8,9,10,11]. The block diagram and the sketch of the signal converting of the interpolation module are shown in Figure 1 and Figure 2. Through this processing, the orthogonal sinusoidal signal is converted into several square-wave signals via the interpolation module. This means that the frequency of the output signal (square wave) is several times higher than the input signal (sinusoidal wave), and the bandwidth of the output signal is based on the clock of the MCU. The cutoff frequency of the output signals is related to the bandwidth of the aforementioned digital processing, and it is the limitation of the moving speed of the target positioning stage. Table 1 shows the relationship between the interpolation factor, the moving speed, and the requirement of the signal bandwidth with an initial resolution of 0.32 μm. A higher resolution requires a higher signal bandwidth, which means that the clock of the MCU and the efficiency of the algorithm are the limitations of the moving speed for the positioning stage. With the commercial interpolation module, people need to make a trade-off between the interpolation factor (resolution) and the moving speed (Table S2, Supplementary Material).

Therefore, in this research, a novel analog interpolation method has been proposed. This method is based on the principle of LIA, and it is designed for the heterodyne laser interferometer. By using the proposed interpolator, the bandwidth of the encoder system can be independent of the resolution. This is an obvious advantage for the encoder system of the next-generation manufacturing equipment.

## 2. Materials and Methods

### 2.1. Laser-Interferometry-Based Encoder

In a feedback-controlled positioning system, an encoder is required as the sensor to provide the positioning information to the controller. An encoder system can be classified into two typical types according to measurement principle and optical structure. One is called a grating-based encoder, and the other is called an interferometry-based encoder. For the grating-based encoder, the initial resolution is based on the grating pitch. Therefore, the initial resolution of the grating-based encoder is about 4 to 40 μm (Table S3, Supplementary Material). On the other hand, the initial resolution of the interferometry-based encoder is based on the laser wavelength and the optical structure [2,12], and its initial resolution is about 0.16 μm or 0.32 μm, depending on its interferometer factor [13]. There have been several times where deviations have existed between the grating-based encoder and the interferometry-based encoder. For the required resolution of the precision positioning stage, the final resolution is around the sub-nanometer scale. The interpolation factors would be over 1000 for the grating-based encoder, and 80 for the interferometry-based encoder. A lower interpolation factor means less processing capacity requirement, and so this is the first advantage of the interferometry-based encoder. Another advantage is the lower interpolation error. Since the interpolation error is highly related to the initial resolution, the interferometry-based encoder has an interpolation error of about a few percent of its initial resolution [14,15]. This is the reason why the advanced precision positioning system is usually built with an interferometry-based encoder [13].

The interferometry-based encoder can be sorted into the homodyne and heterodyne systems according to the laser source. The optical structure and signal processing of these two types of interferometer systems are significantly different [16]. Previous research [17,18] has shown that heterodyne signal processing can have a higher signal-to-noise ratio (SNR), which benefits minimizing the random error of the encoder system. Furthermore, in the multi-axis positioning task, the homodyne interferometer requires more optical elements and detectors to fit the differential signaling [19]. On the contrary, the heterodyne interferometer can share the reference signal, eliminating the need to arrange the reference signal for every positioning axis [6]. This characteristic is advantageous in the positioning application, especially for the multi-axis positioning systems [20,21]. 

**Figure 3:**
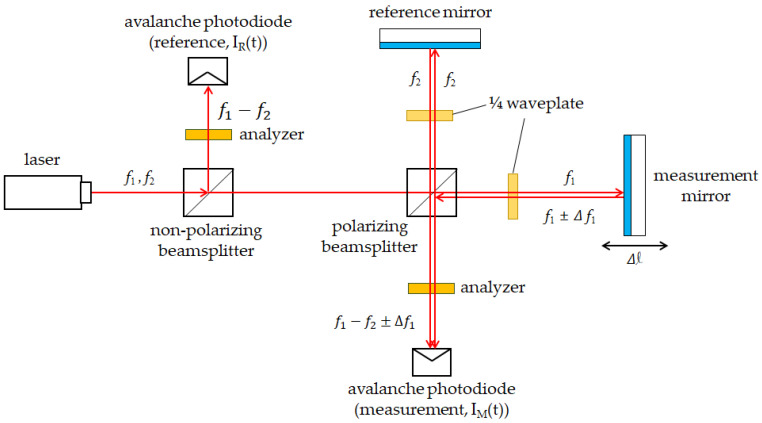
The optical structure of the heterodyne interferometry−based encoder.

Therefore, in this investigation, the target system is the heterodyne interferometry-based encoder, as shown in Figure 3. The interference signal can be expressed as Equations (1)–(4), where $E_{\omega_{1}}\left(t\right)$, $E_{\omega_{2}}\left(t\right)$ and $A_{\omega_{1}}$, $A_{\omega_{2}}$ are the electric fields and amplitude of the two laser beams with difference frequencies; $\omega_{1}$ and $\omega_{2}$ are the angular frequencies of these laser beams; $\phi_{1}$ and $\phi_{2}$ are the phase angles of their optical paths; $I_{R}\left(t\right)$ and $I_{M}\left(t\right)$ are the intensity of the reference and measurement beams; I is the intensity amplitude of these beams; ∆ω is the frequency difference between the two laser beams; and $\theta_{ref}$ and $\theta_{meas}$ are the phase angles of the optical paths within these beams. Because the reference arm ($\theta_{ref}$) is usually fixed, the phase difference between $\theta_{ref}$ and $\theta_{meas}$ can be seen as the displacement information of the measurement mirror. Once we resolve this phase difference (∆∅), the displacement of ∆L can be realized.

(1) $$E_{\omega_{1}}\left(t\right)=A_{\omega_{1}}e^{\left(i\omega_{1}t+\phi_{1}\right)}$$

(2) $$E_{\omega_{2}}\left(t\right)=A_{\omega_{2}}e^{\left(i\omega_{2}t+\phi_{2}\right)}$$

(3) $$I_{R}\left(t\right)=\frac{I}{2}\left[1+\cos\left(∆\omega \cdot t+\theta_{ref}\right)\right]$$

(4) $$I_{M}\left(t\right)=\frac{I}{2}\left[1+\cos\left(∆\omega \cdot t+\theta_{meas}\right)\right]$$

### 2.2. Heterodyne Signal Processing

**Figure 4:**
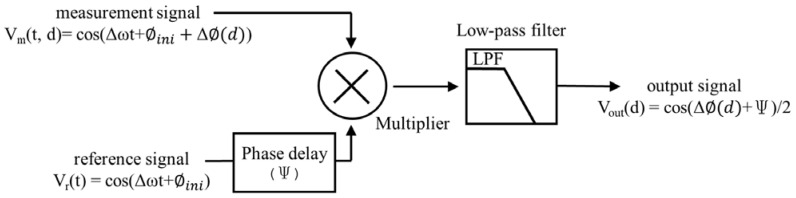
Block diagram of the LIA.

The common heterodyne signal processing is based on LIA technology [22,23], and is wildly used in interferometer signaling [6,24]. The block diagram of the LIA method is shown in Figure 4. The general LIA is composed of a multiplier and a low-pass filter (LPF). The concept of the LIA method is to resolve the phase difference between the two input signals of $V_{R}\left(t\right)$ and $V_{M}\left(t\right)$, as shown in Equations (5)–(7). The phase delay of ψ is the key to signal modulation, and ψ could be any phase angle, e.g., 0, $\frac{\pi }{4}$, $\frac{\pi }{2}$, or $\frac{3\pi }{4}$. The modulation is designed for the quadrature or differential signaling [19,25]. This modulation is recommended for a heterodyne encoder; otherwise, the encoder cannot determine the moving direction, and will be easily influenced by the signal drift.

(5) $$V_{R}\left(t\right)\propto I_{R}\left(t\right)$$

(6) $$V_{M}\left(t\right)\propto I_{M}\left(t\right)$$

(7) $$V_{out}=LPF\left\{V_{R}\left(t\right)\times V_{M}\left(t\right)\right\}\propto \cos\left[\left(∆\emptyset \right)-\psi \right]$$

where $∆\emptyset =\theta_{meas}-\theta_{ref}$.

### 2.3. Interpolation Technology

The interpolator device is a common device that can be found in the motion control system. The function of the interpolator is to enhance the signal resolution and to convert the sinusoidal signal into several square-wave signals. The output signal of the interpolator is called the quadrature-encoded signal. This signal can deal with the digital counter to resolve the direction and displacement of the target stage. The introduction of the digital interpolator and the proposed analog interpolator will follow.

#### 2.3.1. Digital Interpolator

The digital interpolator is usually composed of an input circuit (usually a differential circuit), an ADC, an MCU, and a digital output circuit (usually a comparator circuit) [7,8,9,10,11]. The feature of the interpolator not only enhances the resolution, but also enhances the frequency of the output signal. Therefore, the processing speeds of the ADC and the MCU are the restrictions of the system’s bandwidth. The commercial interpolators are usually designed for the grating-based encoder (Table S4, Supplementary Material). In Equation (8), the maximum velocity can be calculated as 250 mm/s for the grating-based encoder with a grating pitch of 20 μm. Additionally, the maximum velocity of 4 mm/s with the interferometry-based encoder is calculated in Equation (9), where the half wavelength is 0.32 μm. Both of the ADC bandwidths (f) of the interpolator are 125 kHz and the safety factor ($f_{s}$) is 10. When it works with the interferometry-based encoder, the maximum velocity is not enough for a general application, not to mention that the advanced application needs a higher resolution, and so the maximum velocity might be getting worse. For this reason, this investigation would like to find the solution to this problem.

(8) $$V_{max}=\frac{pitch\times f}{f_{s}}=\frac{20\;\mu m\times 125\;kHz}{10}=250\;mm/s$$

(9) Vmax=λ2×ffs=0.32 μm×125 kHz10=4 mm/s

#### 2.3.2. Analog Interpolator Design

**Figure 5:**
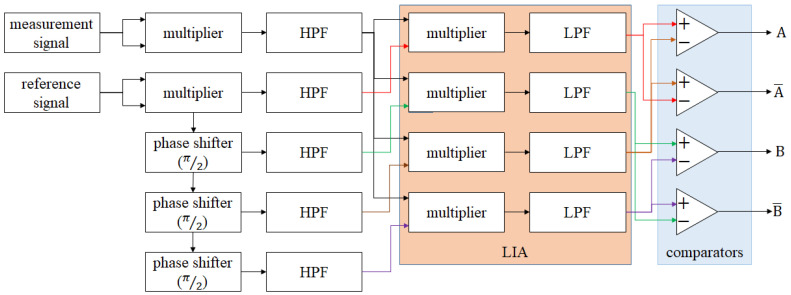
Block diagram of the first-order analog interpolator.

The concept of the analog interpolator comes from the principle of LIA. It uses the multipliers to double the carrying frequencies of the signal. Additionally, it transmits the signal with double frequency to an LIA for resolving the phase difference (∆∅), as shown in Figure 5. In this way, not only does the carrying frequency become two times higher, but also the phase difference becomes double. This means that the sensitivity of the phase difference becomes higher than before. The first half of the derivation of the analog interpolator is shown in Equations (10)–(14). In Equations (10) and (11), the measurement signal and reference signal are multiplied by themselves. Then, the signal modulation (in Equation (7)) is carried out for the frequency-doubled reference signal, and the differential reference signal can be obtained, as shown in Equations (12)–(14) which are the processing of the LIA. After this, we can obtain the information with the doubled phase difference.

(10) $$HPF\left[{cos}^{2}\left(\omega t+\theta_{meas}\right)\right]=HPF\left\{\frac{1}{2}\left[\cos\left(2\omega t+{2\theta }_{meas}\right)+\cos\left(0\right)\right]\right\}=\frac{1}{2}cos(2\omega t+{2\theta }_{meas})$$

(11) $$HPF\left[{cos}^{2}\left(\omega t+\theta_{ref}\right)\right]=HPF\left\{\frac{1}{2}\left[\cos\left(2\omega t+{2\theta }_{ref}\right)+\cos\left(0\right)\right]\right\}=\frac{1}{2}\cos\left(2\omega t+{2\theta }_{ref}\right)$$

(12) $$HPF\left[{cos}^{2}\left(\omega t+\theta_{ref}\right)\right]\to Phase\;shiftingof\frac{n\pi }{2}=\frac{1}{2}\cos\left(2\omega t+{2\theta }_{ref}+\frac{n\pi }{2}\right);n=0,1,2,3$$

(13) $$LPF\left[\frac{1}{2}\cos\left(2\omega t+{2\theta }_{meas}\right)\times \frac{1}{2}\cos\left(2\omega t+{2\theta }_{ref}\right)\right]=\frac{1}{8}LPF\left[\cos\left(4\omega t+{2\theta }_{meas}+{2\theta }_{ref}\right)-\cos\left(2∆\emptyset \right)\right]=\frac{1}{8}cos(2∆\emptyset )$$

(14) $$LPF\left[\frac{1}{2}\cos\left(2\omega t+{2\theta }_{meas}\right)\times \frac{1}{2}\cos\left(2\omega t+{2\theta }_{ref}+\frac{n\pi }{2}\right)\right]=\frac{1}{8}\cos\left(2∆\emptyset +\frac{n\pi }{2}\right);n=0,1,2,3$$

**Figure 6:**
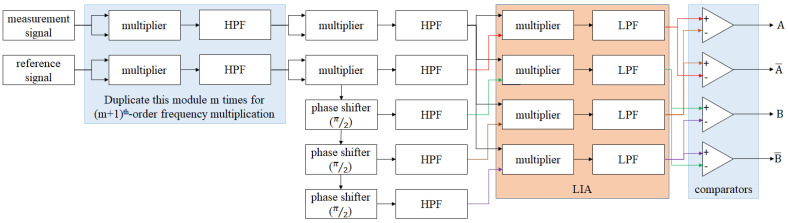
Block diagram of the multi−order analog interpolator.

**Figure 7:**
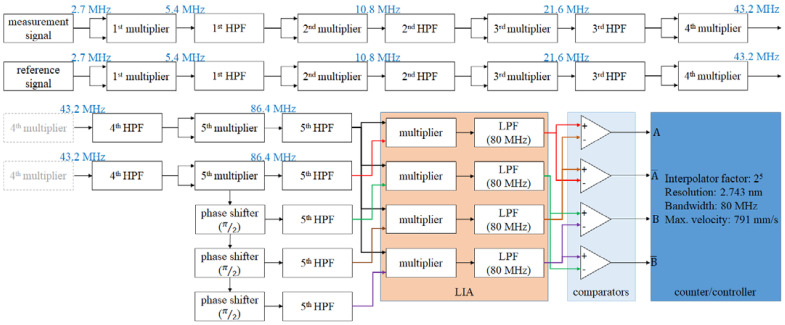
Example: block diagram of 5th−order analog interpolator.

Figure 6 is the block diagram of the multi-order analog interpolator. Additionally, the second half of the derivation of this interpolator is shown in Equations (15)–(17). By duplicating the processing in Equations (10) and (11), the (m + 1)-order interpolator can be realized. For a better understanding of this concept, the example of the 5th-order analog interpolator is shown in Figure 7. The parameters of this example are as follows: the beat frequency of the laser is 2.7 MHz, the wavelength of the laser is 633 nm, the final resolution is 2.473 nm, the bandwidth of the output signal is 80 MHz, and the corresponding maximum velocity is 791 mm/s.

(15) $${\left\{HPF\left[{cos}^{2}\left(\omega t+\theta_{meas}\right)\right]\right\}}^{\left(m+1\right)}=\frac{1}{2^{\left(2^{m}\right)}}\cos\left[2^{\left(m+1\right)}\cdot {(\omega t+\theta }_{meas})\right];m=0,1,2,3,\ldots ,N$$

(16) $${\left\{HPF\left[{cos}^{2}\left(\omega t+\theta_{ref}\right)\right]\right\}}^{\left(m+1\right)}=\frac{1}{2^{\left(2^{m}\right)}}\cos\left[2^{\left(m+1\right)}\cdot {(\omega t+\theta }_{ref})\right];m=0,1,2,3,\ldots ,N$$

(17) $$LPF\left[{\left\{HPF\left[{cos}^{2}\left(\omega t+\theta_{meas}\right)\right]\right\}}^{\left(m+1\right)}\times {\left\{HPF\left[{cos}^{2}\left(\omega t+\theta_{ref}\right)\right]\right\}}^{\left(m+1\right)}\right]=\frac{1}{2^{\left(2*2^{m}+1\right)}}cos(2^{\left(m+1\right)}\cdot ∆\emptyset )$$

## 3. Results

In this investigation, an analog interpolator was tested with a heterodyne interferometric signal. Both the theoretical analysis and experimental result will be described as follows.

### 3.1. Theoretical Analysis

The proposed interpolation method is based on the analog circuit. Different from the digital interpolator, there is no ADC, algorithm, or MUC in the proposed interpolator. Therefore, the bandwidth of the analog interpolator would be the lowest cutoff frequency of these analog circuits, and the lowest one is usually the cutoff frequency of the LPF. To analyze this, Equations (17)–(19) can be obtained. Equation (17) shows the interpolation factor of the (m + 1)^th^-order interpolator, and Equation (17) shows the attenuation factor of it. To summarize the above mentioned discussion, Table 2 shows the planning and specifications of the analog interpolator from the 1st-order to the 10th-order, and the block diagram refers to Figure 6 and Figure 7. In this table, the interpolation factors and attenuation factors are revealed in detail. For the interpolation factors, the final resolution can meet the requirement of the precision positioning stage (Tables S1, S2, and S5, Supplementary Material). For the attenuation factors, it is a disadvantage of the proposed interpolation method. Therefore, we highly recommend adding an automatic gain control (AGC) module or an amplifier after every HPF, as this is an effective solution to this disadvantage. The cutoff frequencies of LPF are designed to be just a little bit lower than the beat frequency or its multiplied frequencies. For the maximum velocities, all of them are the same with the original bandwidth of the laser source. This analysis shows that the converting bandwidth and the maximum velocity of the proposed interpolation method are independent of the resolution and the interpolation factor. 

In addition, the maximum velocity limitation is influenced by both the interpolating resolution and the cutoff frequency of the LPF. The cutoff frequency of the LPF defines the bandwidth of the maximum measurable velocity and serves as a barrier to filter out high−frequency noise. As a result, the cutoff frequency becomes a crucial parameter in the proposed interpolator that needs to strike a balance between the maximum velocity and the SNR. The level of noise is dependent on the encoding system, operational conditions, and environmental factors. Table 2 presents an example of the parameter configurations, which may need to be adjusted according to the specific situation in different applications.

(18) $$interpolation\;factor\;of{\left(m+1\right)}^{th}order\;interpolator=2^{\left(m+1\right)}$$

(19) $$attenuation\;factor\;of{\left(m+1\right)}^{th}orderinter\;polator=2^{\left(2*2^{m}+1\right)}$$

### 3.2. Analog Interpolation Testing

**Figure 8:**
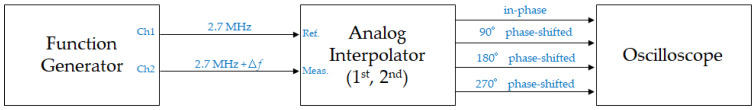
Block diagram of the analog interpolator testing.

To validate the effectiveness of the proposed interpolation method, simulated signal testing was conducted. During the testing, reference and measurement signals with a frequency of 2.7 MHz were generated using a function generator. These signals were then processed using the proposed interpolator, as shown in Figure 8, for both the first and second multiplication orders. The aim of this testing was to validate the effectiveness of the proposed method and, as such, comparators were not included in the system. The generated signals were kept consistent across all tested multiplication orders, and the output signals from the LIA were captured to analyze the signal pattern, frequency, and SNR. To introduce a phase difference between the reference and measurement signals, a slight frequency variation (∆*f*) was added to the signals.

**Figure 9:**
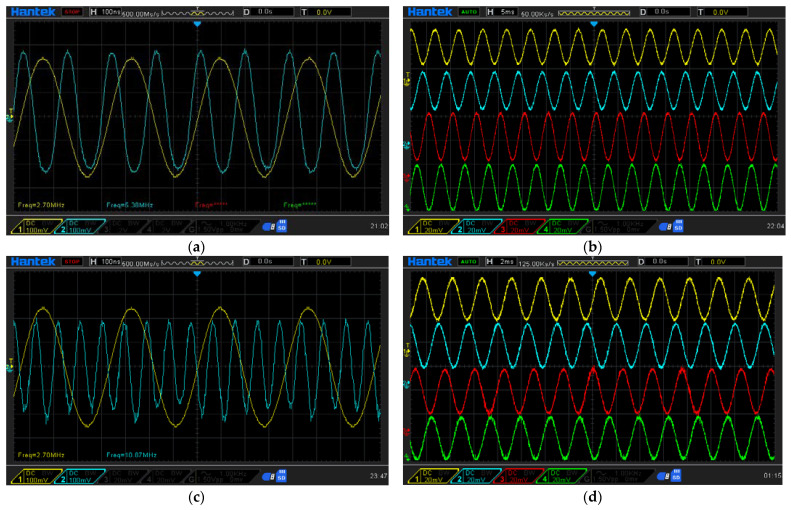
Results of the analog interpolator testing: (**a**) generated signal (blue) and its multiplication signal (yellow) in 1st-order multiplication; (**b**) output signal from the LIA in 1st-order multiplication, vs. in-phase signal (yellow), 90-degree phase-shifted signal (blue), 180-degree phase-shifted signal (red), and 270-degree phase-shifted signal (green); (**c**) generated signal (blue) and its multiplication signal (yellow) in 2nd-order multiplication; (**d**) output signal from the LIA in 2nd-order multiplication, vs. in-phase signal (yellow), 90-degree phase-shifted signal (blue), 180-degree phase-shifted signal (red), and 270-degree phase-shifted signal (green).

The test results indicate that the proposed multiplication method effectively enhances the frequencies of both the reference and measurement signals, as depicted in Figure 9a,c. The SNR values for the first- and second-order multiplications are approximately 20.7 dB and 15.9 dB, respectively, and the resulting encoding signals are stable and compatible with commercial control systems, as shown in Figure 9b,d.

Next, we will analyze the influence of signal attenuation between each order of multiplication and estimate the limitations of the proposed interpolation method. A comparison of the SNR values between the first and second orders reveals a 4.8 dB attenuation in the SNR. Based on previous studies [26,27], the LIA can recover signals with an SNR as low as −24 dB under appropriate conditions. Assuming an SNR attenuation of 4.8 dB with each order of multiplication, the SNR for the 10th-order multiplication is approximately −22.5 dB, which is an acceptable SNR for modern LIA technology. According to Table 2, the 10th-order multiplication can achieve a resolution of 0.077 nm, making it suitable for precision positioning applications.

### 3.3. Experiment Setup

**Figure 10:**
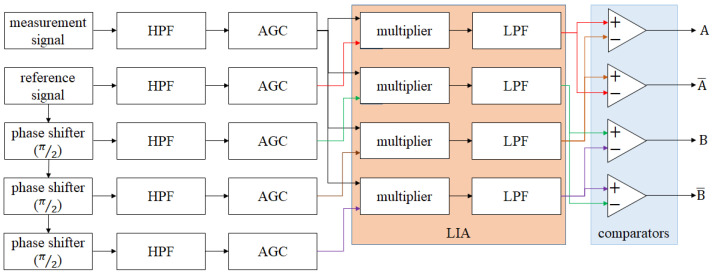
Block diagram of the experimental LIA circuit.

**Figure 11:**
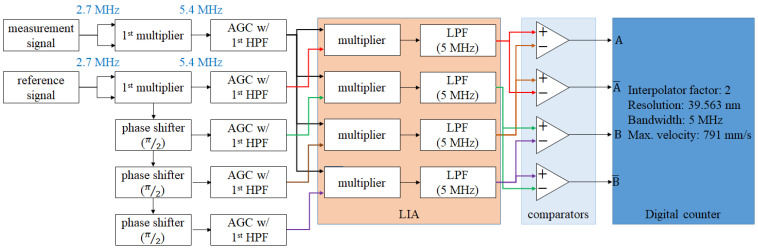
Block diagram of the experimental interpolation circuit.

The experiment setup of the interpolation circuit is shown in Figure 10 and Figure 11. The circuit in Figure 10 is a controlled experimental setup, and Figure 11 is a 1st-order interpolator with an interpolation factor of 2 and resolution of 39.563 nm. The order of magnitude of the resolution is equivalent to the resolution of the commercial laser encoder system (Table S1, Supplementary Material). This interpolation circuit is used to deal with the signal of the heterodyne interferometer with the wavelength of 633 nm, the beat frequency of 2.7 MHz, and an interferometer factor of 2. The specifications of the circuit modules are shown in Table 3, and the experimental result will be shown in the next section.

### 3.4. Experimental Result

**Figure 12:**
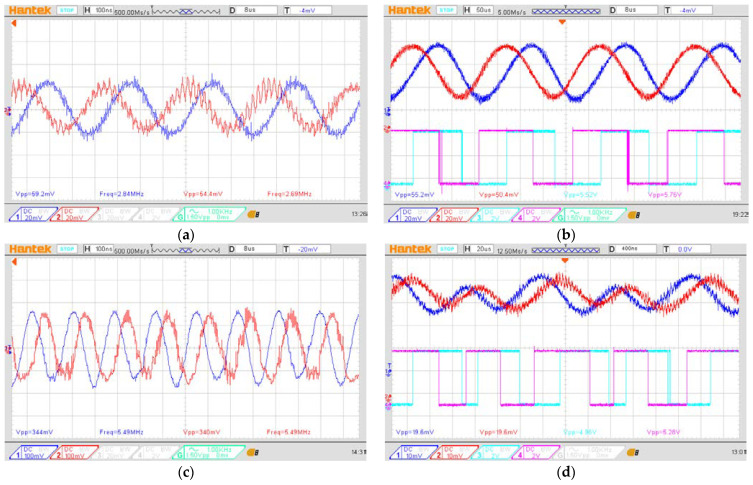
Experimental results: (**a**) the beat frequency of the reference signal (navy blue) and measurement signal (red), (**b**) the encoded signal of the controlled experiment, (**c**) the beat frequency of the reference signal (navy blue) and measurement signal (red) after the 1st-order interpolator, (**d**) the encoded signal from the 1st-order interpolator.

The experimental signal was observed and captured using an oscilloscope. Figure 12a shows the beat frequencies of the reference signal (navy blue) and measurement signal (red), when the measurement mirror is in the static conditions. The detected frequency is around 2.7 MHz, just as with the beat frequency of the laser source. Figure 12b shows the signal passing through the LIA (red and navy blue) and the output signal from the comparators (light blue and purple) with the oscilloscope window width of 50 μs, when the measurement mirror is moving with a constant speed of 2 mm/s. These are the results of the controlled experimental setup, and as we can see it works well. Figure 12c shows the beat frequencies of the reference signal (navy blue) and measurement signal (red) after the first multiplier and in the static conditions. The detected frequency was doubled by the first multiplier, and it is around 5.4 MHz. Figure 12d shows the signal after the LIA (red and navy blue) and the signal from the comparators (light blue and purple) with the oscilloscope window width of 20 μs, when the measurement mirror is moving with a constant speed of 2 mm/s. One can see that the density of the encoded signal of the first−order interpolator is higher than the controlled setup, when under the same moving speed. This means that the interpolator can improve the resolution of the heterodyne signal.

## 4. Discussion

To discuss this research, there are some sections that need to be investigated. First of all is the advantage of the analog interpolation method. The proposed method frees the heterodyne interferometer system from the trade-off between the resolution and the moving speed. The requirements for resolution and accuracy are becoming increasingly strict in modern manufacturing, e.g., the semiconductor industry, photoelectric industry, and panel/display industry. For the advanced positioning device, a resolution of a micrometer or less is inadequate, and thus the need for an interpolator arises. Currently, most interpolation technology is based on digital signal processing. Therefore, the bandwidth of the encoded signal is limited by the clock of the ADC and MCU. Additionally, the complexity of the algorithm which is related to the interpolation factor will affect the converting speed. In view of this problem, a novel analog interpolation method has been proposed in this research. Without the need for digital processing (ADC, algorithm, and MCU), the interpolator’s conversion speed is independent of the interpolation factor. 

Second, the disadvantage of this method and the solution to it will be discussed. Theoretically, the analog interpolator can be stacked multiple times to achieve high-order interpolation. However, the attenuation factor causes the signal to attenuate rapidly. The attenuation factor of the first-order interpolator is 2^3^, and this attenuation is not a serious problem in the beginning. If we place an amplifier after each HPF of the interpolator, this attenuation effect will not accumulate significantly. 

Lastly, the potential of this method should be discussed. Currently, there is rapid growth in the applications of precision positioning stages. Many advanced types of equipment have been proposed. For the technology of the grating-based encoder, the grating pitch is a hard limitation for the positioning accuracy down to the nanometer scale. This presents a great opportunity for the interferometry-based encoders. The interferometry-based encoders have the initial resolution of the sub-micrometer scale, and the speed of light is the definition of the meter. These are the significant advantages of interferometry-based encoders. However, the maximum velocity of the interferometry-based encoders is a limitation for the application field (Table S2, Supplementary Material). For the resolution in the sub-nanometer range, the moving speed may be less than 10 mm/s, and it could be a problem for the production capacity. For this reason, the proposed analog interpolation method could be the solution to this problem. By integrating the analog circuits into an integrated circuit (IC), the interpolator could be a compact module that can be embedded in the sensor head of an interferometry-based encoder.

## 5. Conclusions

In this investigation, a novel analog interpolator was proposed. This system is based on the principle of LIA. It can handle the heterodyne interferometric signal. The output signal is compatible with a commercial motion control system. This interpolation method can be implemented multiple times for a higher interpolation factor. By using this method, there is no longer the need for trade-off between the interpolation factor and the moving speed. It is beneficial for advanced precision positioning applications. 

## Figures and Tables

**Table 1:** Relationship between the interpolation factor, moving speed, and the signal bandwidth.

Interpolation Factor	Moving Speed (mm/s)	Bandwidth (MHz)
1 (resolution of 0.08 μm)	50	0.625
	100	1.25
	200	2.5
4 (resolution of 0.02 μm)	50	2.5
	100	5
	200	10
16 (resolution of 0.005 μm)	50	10
	100	20
	200	40

**Table 2:** Relationship between the multiplication order, interpolation factor, and the resolution.

Multiplication Order	Interpolation Factor	Attenuation Factor	Resolution ^1^ (nm)	Cutoff Frequency of LPF ^2^ (MHz)	Max. Velocity ^3,4^ (mm/s)
None	1	$2^{0}$	79.125	2.5	791
1st	2	$2^{3}$	39.563	5	791
2nd	4	$2^{5}$	19.781	10	791
3rd	8	$2^{9}$	9.891	20	791
4th	16	$2^{17}$	4.945	40	791
5th	32	$2^{33}$	2.473	80	791
6th	64	$2^{65}$	1.236	160	791
7th	128	$2^{129}$	0.618	320	791
8th	256	$2^{257}$	0.309	640	791
9th	512	$2^{513}$	0.155	1280	791
10th	1024	$2^{1025}$	0.077	2560	791

^1^ The resolution is according to the wavelength of 633 nm, the interferometer factor of 2, and quadrature (×4) signal processing. ^2^ The beat frequency of the laser is 2.7 MHz. ^3^ The safety factor (*f_s_*) is set to 1. ^4^ The max. velocities are independent of the resolutions.

**Table 3:** Bandwidth of the circuit module in the experiment.

Circuit Module	Bandwidth
Multiplier	DC—250 MHz
AGC	DC—500 MHz
HPF ^1^	500 kHz (Default)
LPF	1.5 MHz
Phase shifter	2–3.5 MHz
Comparator	DC—200 MHz

^1^ High−pass filter circuit (HPF) is included in AGC circuit (AD8367).

## Data Availability

No new data were created or analyzed in this study. Data sharing is not applicable to this article.

## Supplementary Materials

The following supporting information can be downloaded at https://www.mdpi.com/article/10.3390/mi14030696/s1: Table S1: Specifications of Renishaw RLE series; Table S2: Specifications of Renishaw RLE series with the plane mirror system; Table S3: Specifications of the commercial linear encoder; Table S4: Specifications of the commercial interpolators; Table S5: Specifications of the commercial precision positioning machine.
