# Supplementary material for: A Novel Analog Interpolation Method for Heterodyne Laser Interferometer
Source: Micromachines (Basel). 2023 Mar 21;14(3):696. doi: 10.3390/mi14030696 (PMC10057481; doi:10.3390/mi14030696)
Supplement: Supplementary file 1 [file micromachines-14-00696-s001.zip › micromachines-2243199-supplementary.pdf]

micromachines

MDPI

*Supplementary Materials*

# A Novel Analog Interpolation Method for Heterodyne Laser Interferometer

**Chung-Ping Chang [1,*], Syuan-Cheng Chang [2], Yung-Cheng Wang [2] and Pin-Yi He [2]**

**Table S1.** Specifications of Renishaw RLE series.

| | **Differential System** | **Plane Mirror System** | **Retroreflector System** |
|---|---|---|---|
| Analogue output signal period | 158 nm | | 316 nm |
| Digital quadrature nominal output resolutions | 10, 20, 39.5, 79, 158 and 316 nm | | 20, 39.5, 79, 158, 316 and 633 nm |
| Resolution achievable with REE interpolator | 0.39 nm | | 0.79 nm |
| Resolution achievable with RPI20 parallel interface | 38.6 pm | | 77.2 pm |
| Maximum velocity | <1 m/s | | <2 m/s |

Reference: Renishaw, Data sheet L-9904-2391-04-A.

**Table S2.** Specifications of Renishaw REE series with the Plane mirror system.

| **Model** | **Interpolation factor** | **Resolution (nm)** | **Max. Velocity (mm/sec)** |
|---|---|---|---|
| REE100 | 100 | 3.164 | 35.4 |
| REE200 | 200 | 1.582 | 17.7 |
| REE400 | 400 | 0.791 | 8.8 |

Reference: Renishaw, Data sheet L-9517-9319-01-B.

**Table S3.** Specifications of the commercial linear encoder.

<table>
<tr><th>Suppliers</th><th>Incremental Linear Encoder System</th><th>Accuracy Grade</th><th>Max. Interpolation Error</th><th>Linearity</th><th>Signal Period</th><th>Model</th></tr>
<tr><td rowspan="7">HEIDENHAIN</td><td rowspan="2">Incremental linear measurement with very high repeatability<br>• Steel scale<br>• Small signal period</td><td>± 5 $\mu$m<br>± 3 $\mu$m</td><td rowspan="2">± 0.04 $\mu$m</td><td rowspan="7"></td><td rowspan="2">4 $\mu$m</td><td>LF 485</td></tr>
<tr><td>± 3 $\mu$m<br>± 2 $\mu$m</td><td>LF 185</td></tr>
<tr><td rowspan="4">Incremental linear measurement<br>• Glass scale</td><td rowspan="4">± 5 $\mu$m<br>± 3 $\mu$m</td><td rowspan="4">± 0.2 $\mu$m</td><td rowspan="4">20 $\mu$m</td><td>LS 487</td></tr>
<tr><td>LS 477</td></tr>
<tr><td>LS 187</td></tr>
<tr><td>LS 177</td></tr>
<tr><td>Incremental linear measurement for large measuring lengths<br>• Steel scale tape</td><td>± 5 $\mu$m</td><td>± 0.8 $\mu$m</td><td>40 $\mu$m</td><td>LB 382</td></tr>
<tr><td rowspan="9">RENISHAW</td><td rowspan="6">Incremental linear encoder system : QUANTiC™</td><td rowspan="3">± 5 $\mu$m</td><td rowspan="9"></td><td rowspan="3">± 2.5 $\mu$m</td><td rowspan="6">40 $\mu$m</td><td>RKLC40H-S</td></tr>
<tr><td>RTLC40H</td></tr>
<tr><td>RTLC40H-S</td></tr>
<tr><td rowspan="3">± 15 $\mu$m</td><td>± 3 $\mu$m</td><td>RKLC40-S</td></tr>
<tr><td rowspan="2">± 5 $\mu$m</td><td>RTLC40</td></tr>
<tr><td>RTLC40-S</td></tr>
<tr><td rowspan="3">Incremental linear encoder system : TONiC™</td><td rowspan="3">± 5 $\mu$m</td><td rowspan="3">± 2.5 $\mu$m</td><td rowspan="3">20 $\mu$m</td><td>RTLC20</td></tr>
<tr><td>RTLC20-S</td></tr>
<tr><td>RKLC20-S</td></tr>
</table>

Reference: Heidenhain, Date sheet ID571470.
Renishaw, Website https://www.renishaw.com/en/optical-encoders-installation-guides--31030.

**Table S4.** Specifications of the commercial interpolators.

| Supplier | AMAC ASICs | | iCHaus | |
|---|---|---|---|---|
| Model | IPE 40 | IPE 201 | iC-TW28 | iC-TW8 |
| Interpolation factor | 4–40 | 20–256 | 1–256 | 0.25–16384 |
| Bandwidth | 1.2 MHz–600 kHz | Up to 440 kHz | Up to 700 kHz | Up to 125 kHz |

Reference: AMAC, Data sheet PR-46201-1-4-HB-E-IPE201 & PR-46900-HB-1-3-E-IPE40.
iCHaus, Data sheet TW8_datasheet_C3en & TW28_datasheet_D5en.

**Table S5.** Specifications of the commercial precision positioning machine.

| Technical Data | |
|---|---|
| Measuring and positioning range | 25 mm × 25 mm × 5 mm |
| Resolution | 0.1 nm |
| Probe system | external analog interface for customized probe sensor system is provided |
| Length of the cable between measuring table and electronics unit | approx. 4 m |
| Dimensions (H × W × D) | |
| • NMM-1 | 340 × 420 × 420 mm (without probe sensor) |
| • Electronics unit | 700 × 553 × 600 mm |
| Mass | |
| • NMM-1 | 95 kg |
| • Electronics unit | 75 kg |
| Laser safety class according to EN 60825-1 | 2 M |
| ANSI Z136.1 (CDRH) | II |

Reference: SIOS, Data sheet NMM-1_e_2014_Druck.indd.
